# Data on monthly physicochemical variation of Tropical Island groundwater of Pulau Bidong, South China Sea

**DOI:** 10.1016/j.dib.2020.105527

**Published:** 2020-04-13

**Authors:** Tan Jia Xin, Hasrizal Shaari, Adiana Ghazali, Nor Bakhiah Ibrahim, Kong Sher Rine

**Affiliations:** a Faculty of Science and Marine Environment, Universiti Malaysia Terengganu, 21030 Kuala Nerus, Terengganu, Malaysia; b Institute of Oceanography and Environment, Universiti Malaysia Terengganu, 21030 Kuala Nerus, Terengganu, Malaysia

**Keywords:** Tropical Island, South China Sea, Groundwater water quality, Pulau Bidong

## Abstract

The groundwater samples of Pulau Bidong, Terengganu, Malaysia were collected from five sampling stations from June to October 2016. Physical parameters such as temperature, specific conductivity, dissolved oxygen (DO), pH, salinity, and DO saturation were measured *in-situ* by using handheld device. Meanwhile, total suspended solid (TSS), total dissolved solid (TDS), nitrate (NO_3_^−^), nitrite (NO_2_^−^), ammonium (NH_4_^+^) and phosphate (PO_4_^3−^) were analysed and detected using UV–Vis Spectrophotometer. The inorganic nutrients (NO_3_^−^, NO_2_^−^, NH_4_^+^ and PO_4_^3−^) were ranged from 0.000 to 4.310 mg/L, 0.000 to 0.190 mg/L, 0.000 to 0.807 mg/L and 0.003 to 0.028 mg/L, respectively. The monthly trends of specific conductivity, DO, salinity, DO saturation, NO_3_^−^, NO_2_^−^ and NH_4_^+^ demonstrated significant variation in June (the lowest rainfall) compared to other months. Correlation matrix revealed that temperature was associated with the specific conductivity, and NH_4_^+^ strongly correlated with DO, NO_3_^−^ and NO_2_^−^. Nevertheless, there is a strong negative correlation between physicochemical parameters and monthly rainfall distribution. Notably, future studies are required for long-term monitoring to ensure the good quality of groundwater from Pulau Bidong. The spatial and temporal variability of the present data has been reported by Tan et al. [1].

Specifications Table

**Table:** 

Subject	Water Science and Technology, Analytical Chemistry
Specific subject area	Monthly groundwater quality
Type of data	Table Image
How data were acquired	Physical parameters such as temperature, specific conductivity, dissolved oxygen (DO), pH, salinity, and DO saturation were measured *in-situ* by using Multiparameter YSI Professional Plus. Chemical parameters such as nitrate (NO_3_^−^), nitrite (NO_2_^−^), ammonium (NH_4_^+^) and phosphate (PO_4_^3−^) were measured using ultraviolet spectrophotometric screening method at different wavelengths. Total suspended solid (TSS) and total dissolved solid (TDS) were calculated using gravimetric method.
Data format	Raw Analysed
Parameters for data collection	Physicochemical parameters such as temperature, specific conductivity, dissolved oxygen (DO), pH, salinity, DO saturation, total dissolved solid (TDS), total suspended solid (TSS), nitrate (NO_3_^−^), nitrite (NO_2_^−^), ammonium (NH_4_^+^) and phosphate (PO_4_^3−^) were represented the monthly groundwater variation of Pulau Bidong.
Description of data collection	A total of 25 water samples were collected monthly from five sampling stations between June to October 2016. Groundwater samples were collected in triplicates at each sampling stations. The water level was measured followed by pumping the surface water out from the well for 15 min before taking water samples from each sampling stations. This was done to ensure that the samples collected represent the groundwater instead of the stagnant water, which would affect the quality of the samples. Then, physical parameters of groundwater such as temperature, specific conductivity, dissolved oxygen (DO), pH, salinity, and DO saturation were measured *in-situ* by using Multiparameter YSI Professional Plus. The groundwater samples were later collected using Niskin water sampler and were kept in 1 L and 5 L sampling bottles for nutrients, and total dissolved solid (TDS) and total suspended solid (TSS) analysis. The samples were then preserved in icebox to minimize the changes of nutrients content prior to the laboratory analysis.
Data source location	Institution: Universiti Malaysia Terengganu City/Town/Region: Peninsular Malaysia, Malaysia Country: Malaysia Latitude and longitude (and GPS coordinates) for collected samples/data: Latitude and longitude for each station are presented in the article.
Data accessibility	Data are presented in the article.
Related research article	Author's name: Tan Jia Xin, Hasrizal Shaari, Adiana Ghazali and Nor Bakhiah Ibrahim. Title: Monthly Physicochemical Variation of Tropical Island Groundwater of Pulau Bidong, South China Sea. Journal: Groundwater for Sustainable Development DOI: In Press

## Value of the data

• The researcher can use these data as a baseline data for further investigation of the groundwater quality in the tropical islands.
• Data on monthly physicochemical variation of tropical island groundwater of Pulau Bidong, South China Sea may provide a better picture on the effect of different seasons on the groundwater quality.
• Researchers and Terengganu's stakeholders may use these data for a general overview on groundwater in several islands in Malaysia especially in the East Coast Peninsular Malaysia.
• The present data could complete the environmental data gaps in the South China Sea since no data on groundwater quality in East Coast Peninsular Malaysia.

## 1 Data description

Pulau Bidong is located in the East Coast of Peninsular Malaysia with the total land area of approximately 230km^2^. This island is hilly with steep slope where the maximum elevation is approximately 267 m [1]. The water sources in this island inclusive of small streams in the flat land at the south of the island, which is covered by forest except for the steepest points along the water's edge which are bare rock [2]. Pulau Bidong is an island with tropical rainforest climate under the influence of Northeast monsoon season from October to March. The study area is exposed to heavy rain spells of ∼ 2000 mm annually during Northeast monsoon season [3]. Thus, the main source of groundwater recharge in Pulau Bidong is originated from precipitation. The sampling activities were carried out during June to October as represent the transitional period from Southwest Monsoon to Northeast Monsoon. There are few wells present at both beaches of Universiti Malaysia Terengganu (UMT) Marine Research Station and Pantai Pasir Pengkalan. The wells in Pulau Bidong mainly supply freshwater for various activities and purposes. The sampling points were selected based on the criteria of proper well-constructed at the sampling sites. Three sampling points were located at UMT Marine Research Station and two sampling points were located at Pantai Pasir Pengkalan [1]. The well at station 1 is currently active for freshwater usage, the well at station 2 acts as an alternative freshwater supply and the well at station 3 is inactive, whereas the wells at station 4 and station 5 are mainly used by fisherman for bathing and freshwater source.

Table 1(a)–(h) show the variation of groundwater physico-chemical parameters collected in at all sampling stations in Pulau Bidong. The physico-chemical data were *in-situ* measured using Multiparameter YSI Professional Plus throughout June 2016 to October 2016. The TDS and TSS were measures using gravimetric method after filtration process [1].

**Table 1:** Monthly variation of (a) Temperature, (b) Specific conductivity, (c) DO, (d) pH, (e) Salinity, (f) DO Saturation, (g) TDS, (h) TSS in the groundwater in Pulau Bidong.

a) Temperature ( °C)					

	June	July	August	September	October

Station 1	28.7	28.4	28.2	28.1	27.7
Station 2	28.1	28.0	28.0	27.6	27.8
Station 3	27.6	27.1	27.2	26.9	26.8
Station 4	28.0	27.6	27.6	27.2	26.8
Station 5	27.3	27.3	27.2	26.8	27.2
Average	27.9	27.7	27.6	27.3	27.3
b) Specific Conductivity (S/m)					

	June	July	August	September	October

Station 1	0.293	0.022	0.025	0.010	0.013
Station 2	0.452	0.014	0.021	0.025	0.037
Station 3	0.186	0.009	0.050	0.011	0.012
Station 4	0.080	0.043	0.017	0.015	0.022
Station 5	0.029	0.023	0.018	0.020	0.017
Average	0.208	0.022	0.026	0.016	0.020
c) Dissolved oxygen (mg/L)					

	June	July	August	September	October

Station 1	4.79	1.04	1.11	0.87	2.20
Station 2	3.02	6.40	1.04	0.76	0.32
Station 3	7.45	2.48	0.77	2.27	1.06
Station 4	4.24	0.29	0.26	1.07	1.13
Station 5	5.94	0.48	0.39	1.64	0.27
Average	5.09	2.14	0.71	1.32	1.00
d) pH					

	June	July	August	September	October

Station 1	6.65	5.71	5.43	6.92	5.75
Station 2	6.74	5.78	5.55	6.78	5.98
Station 3	7.23	5.56	5.82	7.07	5.52
Station 4	6.74	5.23	5.50	7.47	6.20
Station 5	5.89	5.98	5.73	7.62	5.57
Average	6.65	5.65	5.61	7.17	5.80
e) Salinity (ppt)					

	June	July	August	September	October

Station 1	1.53	0.10	0.10	0.05	0.06
Station 2	2.41	0.07	0.10	0.12	0.18
Station 3	0.94	0.04	0.24	0.06	0.06
Station 4	0.39	0.20	0.08	0.07	0.10
Station 5	0.39	0.11	0.08	0.10	0.08
Average	1.13	0.10	0.12	0.08	0.10
f) DO saturation (%)					

	June	July	August	September	October

Station 1	48.30	13.40	14.30	11.00	2.20
Station 2	25.90	6.40	13.30	9.70	4.20
Station 3	0.61	31.20	9.30	28.30	1.06
Station 4	41.10	3.60	3.30	13.00	14.20
Station 5	60.60	6.10	4.90	23.00	3.40
Average	35.30	12.14	9.02	17.00	5.01
g) TDS (mg/L)					

	June	July	August	September	October

Station 1	1.662	0.137	1.662	0.086	0.149
Station 2	2.451	0.149	2.451	0.170	0.337
Station 3	1.088	0.093	1.088	0.091	0.160
Station 4	0.450	0.306	0.450	0.125	0.233
Station 5	0.173	0.168	6.840	0.098	0.208
Average	1.165	0.170	2.498	0.114	0.217
h) TSS (mg/L)					

	June	July	August	September	October

Station 1	0.011	0.002	0.003	0.002	0.002
Station 2	0.003	0.001	0.001	0.001	0.001
Station 3	0.003	0.001	0.001	0.001	0.001
Station 4	0.002	0.003	0.002	0.006	0.002
Station 5	0.002	0.001	0.002	0.001	0.001
Average	0.004	0.002	0.002	0.002	0.001

**Fig. 1:**
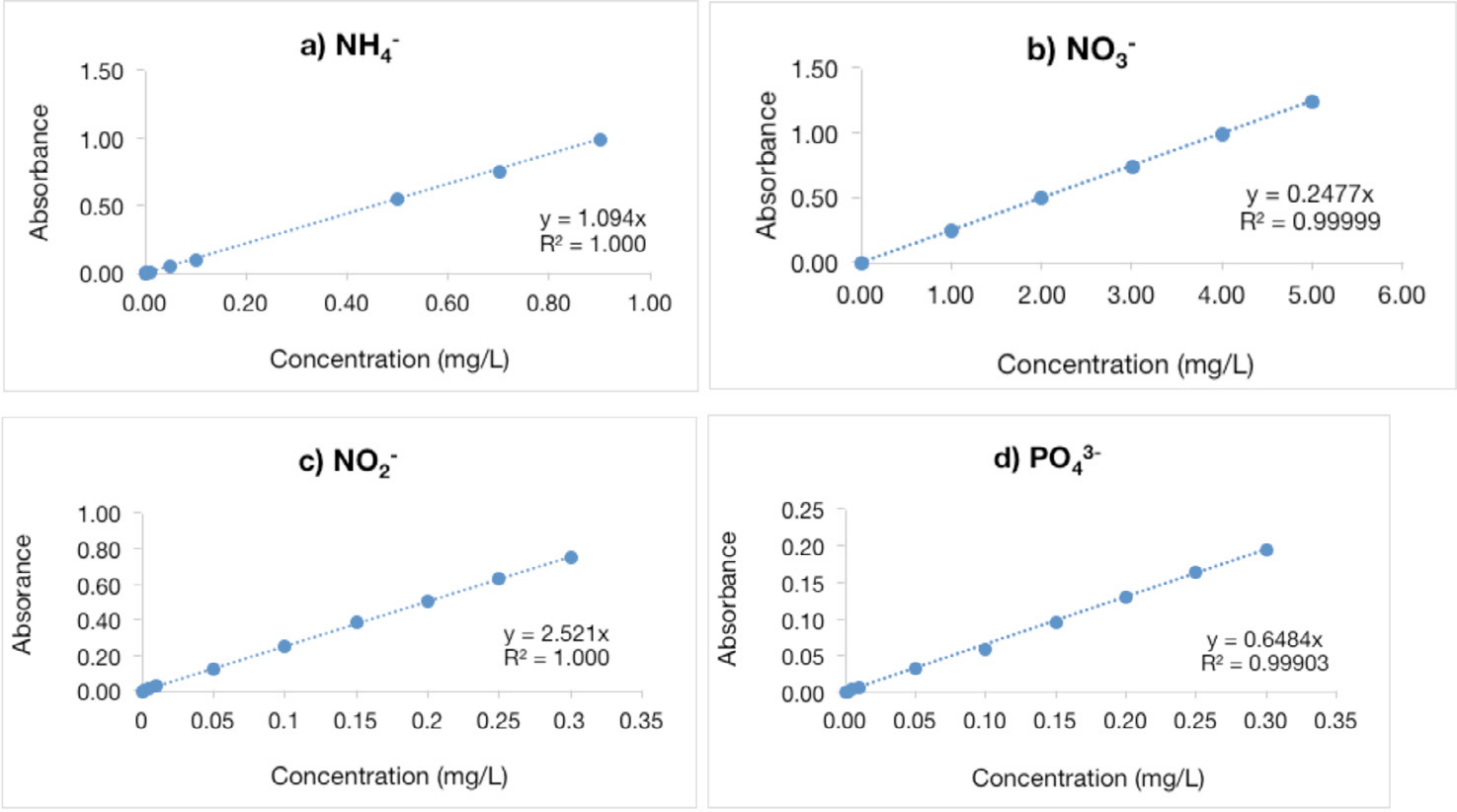
Standard calibration curve for (a) NH_4_^−^, (b) NO_3_^−^, (c) NO_2_^−^ and (d) PO_4_^3−^.

The concentration of nutrients namely ammonium (NH_4_^−^), nitrate (NO_3_^−^), nitrite (NO_2_^−^) and phosphate (PO_4_^3−^) was measured as recommended by American Public Health Association and Environmental Protection Agency standard methods. The concentration of NO_3_^−^ and NO_2_^−^ was determined using ultraviolet spectrophotometric screening method at different wavelengths [1]. In the meantime, PO_4_^3−^ was determined by ascorbic method and NH_4_^+^ was determined by phenate method [1]. Fig. 1 shows the standard calibration curve obtained by ultra violet spectrophotometer throughout the nutrients analysis.

Table 2(a)–(d) show the concentration of nutrients in the groundwater collected at Pulau Bidong during June 2016 to October 2016.

**Table 2:** Monthly variation of (a) NH_4_^−^, (b) NO_3_^−^, (c) NO_2_^−^ and (d) PO_4_^3−^ in the groundwater in Pulau Bidong.

a) NH_4_^+^(mg/L)					

	June	July	August	September	October

Station 1	0.078	0.001	0.001	0.000	0.001
Station 2	0.078	0.005	0.001	0.000	0.004
Station 3	0.807	0.001	0.056	0.000	0.002
Station 4	0.048	0.007	0.005	0.007	0.010
Station 5	0.083	0.025	0.005	0.000	0.040
Average	0.219	0.008	0.014	0.001	0.011

b) NO_3_^−^(mg/L)					
	June	July	August	September	October
Station 1	0.077	0.070	0.085	0.003	0.000
Station 2	0.374	0.000	0.040	0.046	0.023
Station 3	4.192	0.198	4.310	0.421	0.122
Station 4	0.413	0.016	0.031	0.228	0.024
Station 5	0.113	0.546	0.011	0.019	0.011
Average	1.034	0.166	0.895	0.143	0.036

c) NO_2_^−^(mg/L)					
	June	July	August	September	October
Station 1	0.006	0.006	0.005	0.000	0.001
Station 2	0.008	0.003	0.000	0.000	0.003
Station 3	0.190	0.002	0.005	0.001	0.002
Station 4	0.031	0.001	0.004	0.000	0.002
Station 5	0.002	0.009	0.000	0.000	0.000
Average	0.047	0.004	0.003	0.000	0.002

d) PO_4_^3-^ (mg/L)					
	June	July	August	September	October
Station 1	0.004	0.008	0.006	0.004	0.003
Station 2	0.005	0.011	0.008	0.006	0.006
Station 3	0.005	0.006	0.006	0.005	0.005
Station 4	0.007	0.005	0.026	0.028	0.007
Station 5	0.006	0.013	0.006	0.009	0.010
Average	0.005	0.009	0.010	0.010	0.006

## 2 Experimental design, materials, and methods

A total of 25 water samples were collected from five sampling stations between June and October 2016. The water level was measured followed by pumping the surface water out from the well for 15 min before taking water samples from each sampling stations [4]. This was done to ensure that the collected samples represent the groundwater instead of the stagnant water, which would affect the quality of the samples. Water samples were collected using Niskin water sampler and kept in 1 L and 5 L polyethylene bottles. The samples were then preserved in the icebox loaded with ice to minimize the effect on nutrients content prior to the laboratory analysis. The physical parameters such as temperature, specific conductivity, DO, pH, salinity and DO saturation were measured *in-situ* using Multiparameter YSI Professional Plus. The Multiparameter was calibrated according to manufacturer's recommendations to ensure the accuracy of the reading. The concentrations of nitrite (NO_2_^−^), nitrate-nitrogen (NO_3_^−^), ammonium (NH_4_^+^) and phosphate (PO_4_^3−^) were measured according to the standard methods recommended by American Public Health Association [5] and Environmental Protection Agency [6]. The standard solutions were prepared to obtain standard curve and reagents were prepared for the determination of nutrients (NO_3_^−^, NO_2_^−^, NH_4_^+^ and PO_4_^3−^) concentrations in water samples respectively. The nutrients concentrations were determined using ultraviolet spectrophotometric screening method at the different wavelengths for each nutrient. The absorbance values of NO_2_^−^, NO_3_^−^, NH_4_^+^ and PO_4_^3−^ were determined at the wavelengths of 543 nm, 275 nm and 220 nm, 640 nm and 880 nm. There are two wavelength values were used for the NO_3_^−^ analysis (220 and 275 nm) compared to NO_2_^−^, NH_4_^+^and PO_4_^−^. The absorbance values at the wavelength of 275 nm were subtracted from the absorbance value at the wavelength of 220 nm in order to discard the interference of dissolved organic matter. The TDS and TSS concentrations in the sample were determined using gravimetric method [5]. Water samples were filtered through GF/C filter paper by using filtration set. The filter paper was transferred into the petri dish and dried for an hour at 103 °C. Then, the filter paper was cooled down in desiccator for an hour and weighted using analytical balance. The TSS value in samples was calculated based on the following equation [6]:

TSS(mg/L)=(A−B)×(1000/v)

Where,

A = Initial weight of filter paper + dried residue (mg)
B = Initial weight of filter paper (mg)
v = The volume of water sample (mL)

250 mL filtered water samples were transferred into glass beakers in three replicates. The beaker were heated in oven at 103 °C until water samples completely evaporated. The beaker was cooled down in desiccator for an hour, and the weight of beaker was measured using analytical balance. The readings were recorded until weight of the beaker constant. The TDS value in water sample was calculated based on the following equation [5]:

TDS(mg/L)=(A−B)×(1000/v)

Where,

A = Initial weight of beaker + dried residue (mg)
B = Initial weight of beaker (mg)
v = The volume of water sample (mL)
